# EAC-1A: A novel large-volume lunar regolith simulant

**DOI:** 10.1038/s41598-020-62312-4

**Published:** 2020-03-25

**Authors:** V. S. Engelschiøn, S. R. Eriksson, A. Cowley, M. Fateri, A. Meurisse, U. Kueppers, M. Sperl

**Affiliations:** 1 The Natural History Museum, University of Oslo, Oslo, Norway; 2 European Astronaut Centre EAC, European Space Agency, 51170 Köln, Germany; 3 Institute for Materials Physics in Space, German Aerospace Center (DLR), 51147 Köln, Germany; 4 Department of Earth and Environmental Sciences, Ludwig-Maximilians-Universität (LMU), Munich 80333 München, Germany

**Keywords:** Ceramics, Mineralogy, Mechanical properties

## Abstract

The European Astronaut Centre (EAC) is currently constructing the European Lunar Exploration Laboratory (LUNA), a large training and operations facility to be located adjacent to EAC at the DLR (German Aerospace Centre) campus in Cologne, Germany. With an estimated representative lunar testbed area of approximately 660 m^2^, a large volume of lunar regolith simulant material is needed for this purpose. In this study, a basanitic sandy silt from a quarry located in the Siebengebirge Volcanic Field is evaluated as a large-volume source of material. The focus of this project has been to conduct a physical and chemical characterisation of the fine-grained material to be used in LUNA; the European Astronaut Centre lunar regolith simulant 1 (EAC-1 A). The physical characterisation tests undertaken include sphericity, density measurements, cohesion and static angle of repose, with mineralogical investigations via petrographical analysis with optical microscope and SEM, XRF, XRD and DSC measurements. The results of the EAC-1A tests are compared to published data on existing widely used lunar regolith simulants, namely JSC-1A, JSC-2A, NU-LHT-3M, DNA and FJS-1.

## Introduction

It is foreseen that the International Space Station (ISS) program will come to an end in the mid-to-late 2020’s, and as such, many space agencies are looking towards destinations beyond low earth orbit (LEO) in order to further manned exploration of our solar system. The Moon is our closest planetary neighbour and is therefore viewed as a logical next target for future human exploration^1^. Due to the Moon’s close proximity to the Earth, it has long featured as a stepping stone on the journey towards human exploration on Mars and is now widely reflected as such in international exploration roadmaps (for example, see the latest International Space Exploration Coordination (ISECG) Global Exploration Roadmap). Lunar samples returned on the Apollo 11–17 and three Soviet Union missions (LUNA 16, 20 and 24), in addition to the variety of surveyor probes and rovers sent there, have yielded a vast reservoir of scientific knowledge on the Moon and our Solar System. The scientific output of returning to the lunar surface is also of major consideration, and it is widely acknowledged that answering current questions about the Moon will help us understand other terrestrial bodies in the Solar System^1^. In the context of likely future human and robotic exploration of the lunar surface, ESA has begun the development of the European Lunar Exploration Laboratory (LUNA) at EAC, Cologne, Germany. This facility will address the growing needs of Europe to test, validate and develop technologies relevant for lunar exploration as well as practice surface operations to prepare for possible renewed human missions to the Moon. The LUNA testbed will consist of two areas; a main testbed area of approximately 660 m^2^ for surface operations testing, and a to-be-further-defined regolith simulant ‘dust’ chamber of approximately 50 m^2^.

A homogenously basanitic material, dubbed “EAC-1”, has been identified to be used in LUNA and as a large volume baseline simulant. The lunar regolith simulant EAC-1 is quarried from an intracontinental basanite province located in Siebengebirge Volcanic Field, Königswinter, Germany^2^. The mechanically crushed basanite is produced by *Rheinische Provinzial-Basalt- u. Lavawerke GmbH & Co. oHG* (RPBL) in Königswinter, Germany. The material can be provided in bulk in five different grain size ranges, of which three have been considered of interest in this study; 0.02–0.2 mm, 0.2–0.5 mm and 0.5–1.0 mm. Where the properties of the dusty simulant have been measured, the figures are labelled with “EAC-1A”. For non-grainsize dependent tests where samples of the Königswinter basanite rock specimens have been used, these are simply labelled as EAC-1. This does not refer to a specific grain size, but to the material itself.

A lunar simulant is defined as “a granular or powder material that mimics one or more properties of the material found on the Moon”^3^. Lunar regolith is a mixture of fragments of rocks, glass, agglutinates (welded aggregates) and breccias (broken rock fragments cemented together), created through space weathering and meteorite impacts^4,5^. Simulant materials are needed to test every operational aspect that requires contact with lunar regolith, and understanding the behaviour of the lunar regolith is of crucial importance in order to traverse and to utilize the native resources available on the Moon through *In-Situ* Resource Utilisation (ISRU)^6^. Consequently, a variety of lunar regolith simulants (such as JSC-1, FJS-1 and CAS-1) have been commercially produced and are widely used for lunar research^3,7^. Other engineering lunar regolith simulants developed for similar purposes are for example GRC-1^8^ and TJ-1^9^.

Consideration must always be given to the correct application of simulants – no singular regolith simulant can cover the multitude of use cases that lunar regolith involves^7^. Additionally, the simulant materials are in many cases produced for a specific cause, without a published comprehensive characterisation or description^7^. In 2010, the National Aeronautics and Space Administration (NASA) developed Figures of Merit (FoM) in order to efficiently characterise and classify simulant materials^10^. However, with the cancellation of The Constellation Programme (CxP), activities in the area decreased and there is currently no internationally accepted classification scheme for simulant materials.

Lunar regolith simulants are commonly produced in small quantities, insufficient for the large volume needs of the LUNA facility. The constraints of sourcing a simulant for LUNA has therefore been to identify a material (1) available in quantities between 600–1000 tonnes, (2) of <1 mm grain size, (3) at a low production and transportation cost and (4) of a composition and nature that is acceptable as a lunar regolith simulant. Five different commonly used simulants were selected for comparison to EAC-1A due to their accessibility; JSC-1A, JSC-2A, DNA, FJS-1 and NU-LHT-3M. Additional simulants have also been included for comparison where values are available from literature. JSC-1A was produced by Orbitec on behalf of NASA (replicating JSC-1, developed at the Johnson Space Center), JSC-2A and NU-LHT-3M, both by Zybec Advanced Products Inc., DNA, created by Monolite for an ESA GSP study^11^, and FJS-1, produced by Shimizu Corp. for JAXA (then: NASDA)^12^. A chemical comparison was also made to the Apollo regolith samples as published online by the Lunar and Planetary Institute (LPI). Herein, we provide a characterisation of the chosen material, describing the physical and chemical properties for future users of LUNA.

## Results

### Grain size distribution

**Figure 1:**
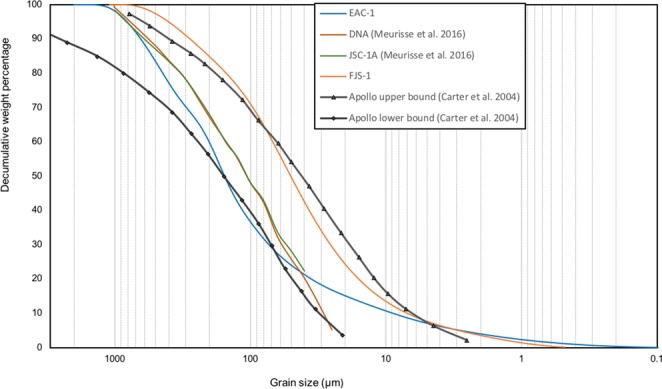
Grain size distribution curve. The grain size distribution of EAC-1A, DNA and JSC-1A, plotted together with the bulk grain size range of the Apollo samples^13^.

The grain size distribution of EAC-1A was analysed and compared to the bulk range of the lunar regolith grain size distribution (Fig. 1)^13^. Grain size distribution was measured by using a Beckman Coulter LS 13 320 Laser Diffraction Particle Size Analyzer. The sorting grade of EAC-1A is poor, which means there is a wide range of grain sizes present in the material. Following the Wentworth classification scheme, EAC-1A is classified as a silty sand^14^. The various lunar regolith samples do, not surprisingly, show an overall wider range; from silty sand to sandy silt^13^. Grain size distribution measurements of the lunar regolith simulants DNA and JSC-1 show that these are modestly sorted and can be classified as sandy silts.

In order to effectively compare the very smallest with the biggest grains, the unit φ is often used, which is a logarithmic conversion of the grainsize (−Log^2d^, where d = diameter). The grain sizes in EAC-1A span from 14.6 φ (clay fraction) to −1 φ (very coarse sand), with a median value of 6.47 φ (~11 µm). In comparison, the Apollo samples range in median grain size values from 4.58 φ (42 µm) to 3.40 φ (94 µm) (Apollo 12), to 3.74 φ (75 µm) to 0.32 φ (802 µm) (Apollo 14)^4^. EAC-1A consequently appears to contain more fines, however, a proper comparison is hindered in the <10 µm fraction by the impracticality of getting exact data for this fraction during the sieving of the Apollo samples^15^.The distribution of the EAC-1A grain sizes mainly lie within the upper and lower Apollo boundary, although EAC-1A has a slightly lower weight percent of particles between ~180 µm and 70 µm than the lower limit of the bulk Apollo lunar regolith samples^13^.

### Density

Before measuring the density, the water content was measured by heating and drying EAC-1A in air, following the ASTM D4442 Standard. This procedure released ~0.8 wt% water from the simulant. Absolute density was measured using a gas pycnometer Micromeritic AccuPyc II. Bulk density was measured by weighing a specific volume of the simulant.

The bulk density (density of material at optimal packing) of EAC-1A, JSC-1A, JSC-2A, DNA and NU-LHT-3M was measured, resulting in 1.45, 1.56, 1.44, 1.27 and 1.54 g/cm^3^ respectively. These values are comparable to what was measured for the lunar regolith using drill cores during the Apollo missions, with a surface density of 1.30 g/cm^3^, quickly increasing to 1.52 g/cm^3^ then gradually to 1.83 g/cm up to 100 cm depth^16,17^. The absolute density measurements (volume measurement excluding pore space) of EAC-1, JSC-1A, JSC-2A, DNA and NU-LHT-3M resulted in 2.90, 2.92, 2.89, 2.79 and 3.08 g/cm^3^ respectively. As the results have shown, JSC-1A and EAC-1A have the highest bulk and absolute densities among the measured samples.

### Cohesion

Terrestrial materials typically have no to very little apparent macroscopic cohesion, where the physical origin of cohesion is either formed by capillary forces or from the electrostatic bonds between clay and silt particles^18^. The lunar regolith is generally more cohesive than terrestrial materials due to the interlocking of the irregular, sometimes re-entrant surfaces of the grains, as they have not been subject to terrestrial, physical weathering processes^17^, and the lack of adsorbed gases that coats and lubricates the particles^19^. The cohesion of lunar regolith is estimated to range from 0.1–1.0 kPa on average, and is increasing with higher density (decreasing porosity)^20^. The typical range is 0.44–0.62 kPa in the upper 15 cm of the regolith surface and down to 2.4–3.8 kPa in the lower 30–60 cm^21^. JSC-1A also has a linear relationship, ranging from 0.0–1.0 kPa with increasing densities from 1.62–1.96 g/cm^3,22^. The cohesion of EAC-1A at a density of 1.95 g/cm^3^ was estimated to be 0.38 kPa, however, it was not possible to establish the relationship between density and cohesion. FJS-1 has a cohesion of 8 kPa although it is not stated at which density^23^, however, studies have investigated the slope angle and self-standing heights^24,25^. There are no published data on the cohesion of DNA or NU-LHT-3M, but it is considered to be negligible in NU-LHT-2M^26^. In this study cohesion was measured by lowering a plastic plate into a box filled with EAC-1A. The measurements were repeated 8 times for different densities. In order to increase the density, the sample was placed in a shaking machine (amplitude 20).

### Sphericity

In total 80 particles were selected for the sphericity study. The sphericity measurements were done manually using a Zeiss Axio Imager A2 optical microscope. The calculations to determine the sphericity were based on the method developed by Krumbein^27^. The analysed particles were from the two larger grain size factions in the EAC-1 range; 0.2–0.5 mm and 0.5–1 mm. The particle size range of 0.02–0.2 mm proved to be too small in order to make accurate measurements from visual observations. The average sphericity for the grain size fraction of 0.2–0.5 mm was 0.601 Φ and 0.591 Φ for the 0.5–1 mm fraction. Both of the measured grain size fractions of EAC-1 display a wide range of shapes; from elongated, spherical to platy particles, while lunar regolith grains are extremely irregular, often re-entrant and tend to be slightly elongated (average elongation of 1.35)^17^. Other features of the grain shape such as the aspect, the elongation, the angularity or the volume ratio to ellipsoid were also reported by Katagiri *et al*.^28^. The determination of these parameters requires the use of X-ray CT and advanced image processing and was not available in this study.

### Mineralogical analysis

#### Thin sections

**Figure 2:**
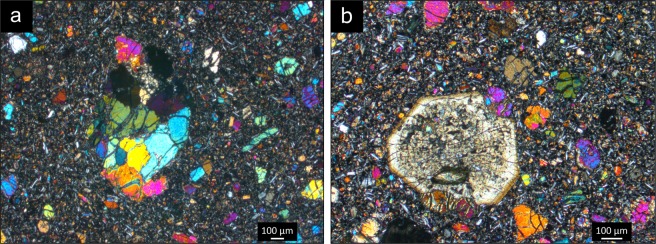
Thin sections under polarised light. To the left, the glomerocryst-containing unit, showing an olivine glomerocryst. On the right, a large pyroxene crystal from the aphanitic unit. Note the similar feldspar and olivine-rich, fine-grained matrix in both units.

The thin sections were prepared at the Ludwig-Maximilians-Universität (LMU) Munich. Each sample was cut and glued to a glass surface before polishing to 30 µm. The samples were analysed in polarised microscope for mineral identification and mineral volume percentage determination. Two mineralogical units were identified from thin section analysis of the Königswinter basanite (Fig. 2); a homogenously aphanitic unit, and a unit with an aphanitic matrix containing olivine glomerocrysts (~1 cm) and larger xenoliths (>10 cm). The xenoliths are peridotites which, based on visual observations of the hand specimens, range from dunite (>90% olivine) to olivine-rich lherzorite (~40–90% olivine). The major minerals are plagioclase, olivine and pyroxene, with ~10% pyroxene in both units, slightly higher olivine content (~30%) in the glomerocryst-containing unit than in the aphanitic unit (~ 25%), while the latter has a higher plagioclase content (~65%) than the former (~55%). Plagioclase is almost exclusively present among the <100 µm crystals. Primary, euhedral olivine crystals span from ~20 µm to ~500 µm, with the olivine glomerocrysts being significantly larger (~600–1300 µm). Pyroxene crystals are euhedral with a size range between 100–600 µm. Amphibole and quartz are present as minor minerals. Quartz crystals are sparse, but they occur as angular xenocrysts with clear reaction rims consisting of plagioclase, olivine and pyroxene in the glomerocryst unit, and as anhedral inclusions in the aphanitic unit. The amphiboles are between ~100–200 µm and are euhedral and without reaction rims. The aphanitic unit sample contains an indeterminate fibrous mineral cluster, which could be chlorite. A clear reaction rim of plagioclase and pyroxene encloses the fibrous glomerocryst. No petrographic fabric is present in any of the samples.

### XRD

X-Ray Diffraction (XRD) analysis was performed on solid rock samples from the two basanite units of EAC-1; the aphanitic unit and the glomerocryst-containing unit, and on the EAC-1A simulant. Both units contain fosterite, fayalite, Ca- plagioclase, diopside, augite and titanite, with possible nepheline. The aphanitic unit also show possible traces of clinochlore and additional feldspathoid peaks. The XRD data obtained from EAC-1A is similar to the two basanite units, but with weaker ilmenite and titanite signatures. Characteristic XRD scans can be seen in Supplemental Fig. 1. The mineral identification is based on X-ray diffractogram comparison to reference mineral data given by the RRUFF database (2017). Wet polishing of the basanite hand specimens were performed using Schmitz-Metallographie GmbH Silicon Carbide Grinding Papers (500 and 800 micron). One alteration mineral region was selected for analysis. The later were prepared in the same manner as the olivine rich and olivine poor samples. Additionally, powder XRD were acquired from EAC-1. The Siemens D5000 Powder diffractometer with a wavelength of 15 406 nm was used for all XRD tests. The samples were 1–2 mm thick with 18 mm diameter. The step scan was set to a range between 10 to 100°. Measurements were taken with 0.02° steps of 800 μm with a 6 second acquisition time.

### XRF

X-ray fluorescence analysis (XRF) has been used to perform geochemical analysis of two representative samples. To this end, 3 g of powdered sampled have been heated in a muffle furnace to 110 °C (6 h for drying, i.e. release of adsorbed water) and subsequently to 1050 °C (2 h for release of chemically dissolved water, i.e. loss on ignition, LOI). Of the remaining powder, 1 g has been mixed with 9 g of chemical agent (Dilithiumtetraborat, Li_2_B_4_O_7_) to produce a glass tablet following melting and quenching. The glass tablet was subsequently measured in a Philips MagiX Pro WD-XRF device to reveal major and trace elements.

**Figure 3:**
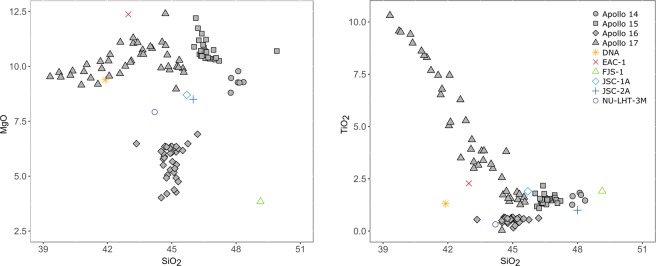
Major element compositions. The MgO and TiO_2_ compositions of the studied lunar regolith simulants are compared to samples taken during the Apollo 14, 15, 16 and 17 missions. EAC-1 values fall within the range of the Apollo 17 samples.

**Figure 4:**
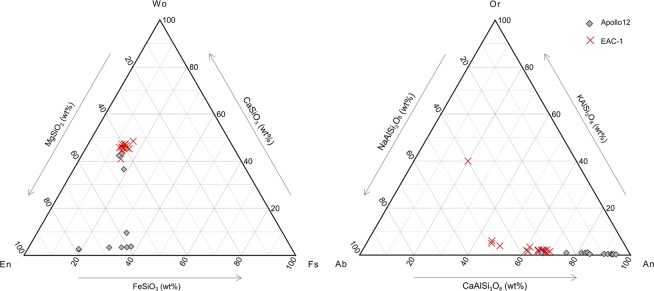
Total-alkali-silica diagram. NaO_2_ and K_2_O plotted against SiO_2_ illustrates how the lunar samples contain much less alkali minerals than terrestrial materials. The only lunar regolith simulant with a comparable alkali content is NAO-1^32^.

EAC-1 contains 11.9 wt% MgO and 2.4 wt% TiO_2_ (Fig. 3). EAC-1 contains 43.7 wt% SiO_2_ and 4.2 wt% Na_2_O + K_2_O, placing it within the lower range of the basanite field of the total alkali-silica (TAS) diagram. The alkaline bulk composition differentiates EAC-1 from the lunar regolith, which ranges between 1.32 wt% to 0.4 wt% - considerably lower compared to the geological material found on Earth (see Figs. 3 and 4)^29^. EAC-1A is most similar to the intermediate compositions of the Apollo 17 samples^30,31^. Lunar regolith simulants such as JSC-1A, JSC-2A, TJ-1, MLS and DNA are alkali-rich basalts and basanites. The simulant that lies closest to the lunar regolith in alkali composition is NAO-1, which originates from a basalt with an alkali content of <1 wt%^32^.

**Figure 5:**
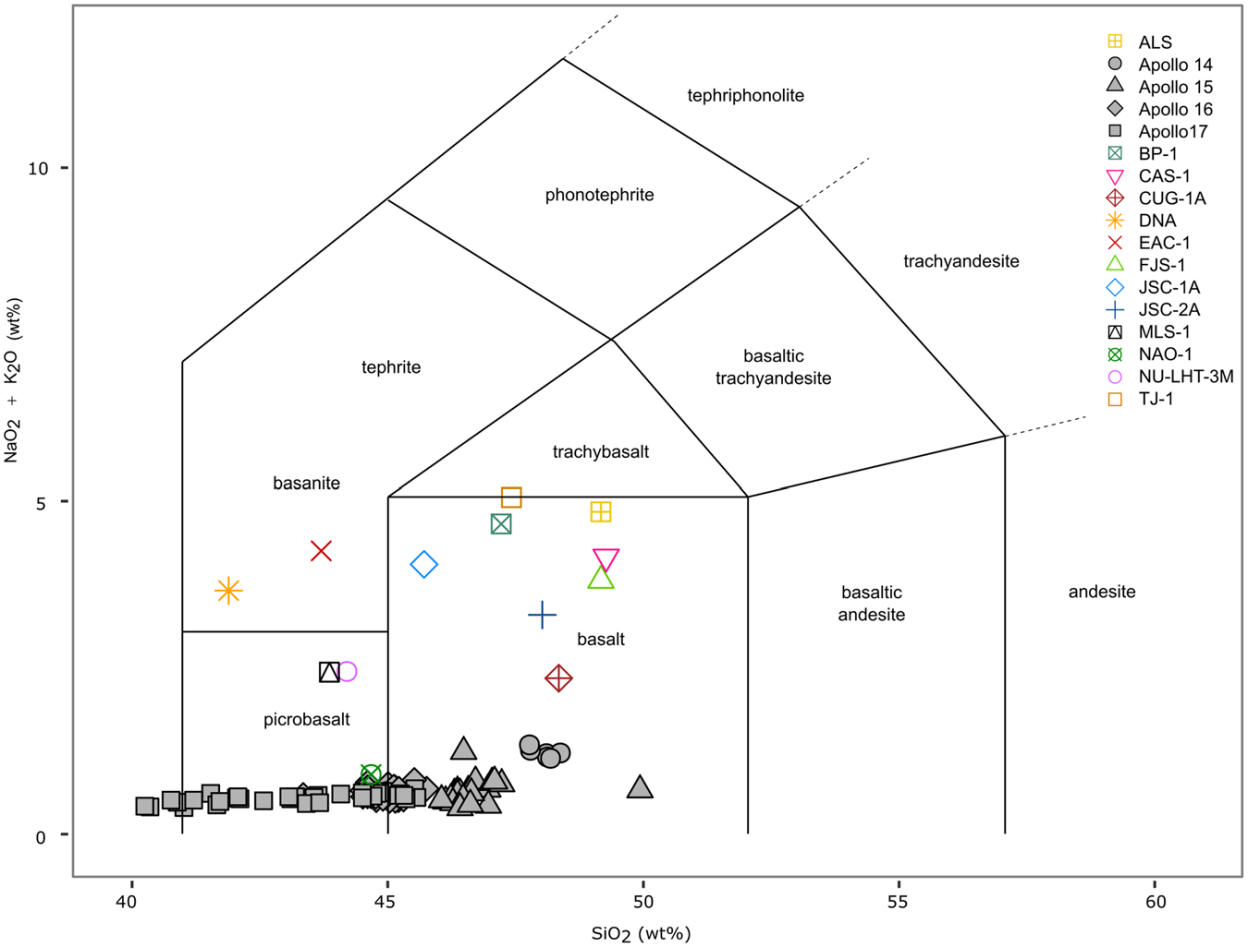
Ternary diagram for the plagioclase and pyroxene endmember compositions. EAC-1 compared to Apollo 12 samples.

The endmember composition of the major mineral constituents were analysed using energy-dispersive X-ray spectroscopy (EDX) during Scanning Electron Microscope (SEM) imaging and compared to Apollo 12 data (Fig. 5). The SEM used for the chemical characterization of EAC-1 was a Zeiss Ultra 55 scanning electron microscope. Resolution ranging between 1–2 nm with an accelerating voltage of 15 kV. Systems used were Oxford INCA EDX and Oxford Channel5/NordlysII EBSD. During the analysis the running time was set to 50 second with a counting rate of 2.65 Kc/s. The dead time was below 30% during all measurements. One olivine – poor (OP-1) sample and one olivine rich (OR-1) sample were analysed. Point measurements were carried out at three different locations including three unique particles ranging in grain size from 50–300 μm. (FeatureScan software). A representative SEM image and EDX analysis are presented in the Supplemental Information (fig. x).

The lunar regolith is dominated by anorthite; the high-Ca wt% plagioclase endmember. EAC-1 also contains high Ca wt% plagioclase, however, generally lower than in the lunar regolith (~50–70 wt% as compared to ~65–95 wt%). The pyroxenes found in EAC-1 are mainly Mg-rich augite, and no enstatite compositions are present. The Apollo 12 samples, however, include both enstatite and augite components.

### DSC

**Figure 6:**
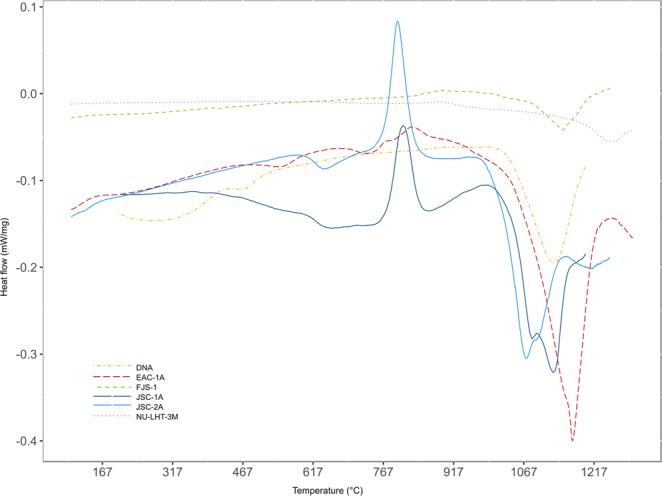
Differential scanning calorimetry measurement. The lunar regolith simulants were heated stepwise to determine whether any glass phase was present in the material, and to find the melting point.

Differential Scanning Calorimetry (DSC) measurements were performed on six lunar regolith simulants, including EAC-1. DSC provide information regarding the glass content in the simulants, and the exothermic peak marks the melting point of the materials and glass melts at a lower temperature. Measurements were carried out under argon atmosphere with a Pegasus DSC 404 C. The lunar regolith simulants were put in 5 mm diameter and 5 mm high crucibles, which were heated up by 10 K/minute from room temperature to 1,200 °C. EAC-1, NU-LHT-3M and FJS-1 are fully crystallised materials (Fig. 6), as is DNA^33^. They display a prominent endothermic peak between 1080 °C to 1250 °C, but no major exothermic transformations. In contrast, the glass content of JSC-1A is around 50%^34^, and both JSC-1A and JSC-2A have glass transition peaks. For both materials, the glass transition (Tg) is 620 °C, while the crystallisation peak (Tc) occur at 800 °C. Lunar glass is formed by volcanism and meteorite bombardment (impact melting), and as a result of the scale of these processes, lunar rocks can occur as a range from fully crystalline to completely glassy^4^. The properties of lunar glass are difficult to mimic on Earth, and influence the melting properties and reactivity of the material^35^.

## Discussion

To develop technology at higher technology readiness levels (TRL) and in order to test more challenging and realistic scenarios, a dusty lunar regolith simulant is needed. However, a coarser, dust-free version of the EAC-1 host material will be used for the main lunar regolith testbed to allow for easier operations training and earlier stage hardware testing. The details of the LUNA dust chamber are yet to be defined following the construction of the main LUNA test bed in 2021 Currently, EAC-1A is already taken in use for small-scale, low-TRL experiments undertaken by ESA. This included testing for the ESA PROSPECT activity, which will drill the lunar surface in a future Roscosmos-ESA joint mission. The simulant is also currently in use by other institutions at various sites, for example investigating regolith derived ceramic coating via deposition processes^36^, the production of glass and mirrors from simulant^37^ as well as used for thermal analysis and ISRU processing studies. The material is also stored and curated by the ESA Sample Analogue Curation facility at ECSAT, UK^38^.

Simulants can never fully imitate all the intrinsic properties found in the actual lunar regolith due to the differences between the terrestrial and lunar environment. The properties of interest therefore have to be carefully selected, and simulant usage properly considered in the context of their proposed application. In addition, factors such as homogeneity of the material, reproducibility (i.e. quality control from batch to batch), price and logistical availability, are also important for the use of a given material for future studies. This study aims at providing an overview of the physical properties of EAC-1A and the chemical and mineralogical characteristics of the host material EAC-1, in order for potential future users of the material and the LUNA facility to evaluate whether the simulant presented herein meets their needs. The focus has been to provide a comparison to other commercially available regolith simulants and not only the lunar regolith, as simulant material is what would available to most users of both the LUNA facility and EAC-1A.

From the physical properties tests it is shown that the grainsize distribution lies within the bulk range of the grain size of the Apollo samples^13^, however, the median grain size of EAC-1A is higher than what has been reported the lunar regolith (~70 µm or ~40–130 µm range)^13^. EAC-1A does not show any sphericity trends, which is expected in a granular material produced by blasting and crushing rocks into small grain sizes. The widely dispersed variety of shapes is similar to lunar regolith, however, EAC-1A does not have as highly irregular, re-entrant, angular or even spherical grain surfaces as the lunar regolith, which is a unique feature of space weathering^17,21^. Based on preliminary measurements, EAC-1A appears to have a lower cohesion than JSC-1A, and much lower than reported for FJS-1, but this requires further study. However, direct comparison to the literature is not possible due to the different characterisation methods used, possible variations in moisture content, and in the particle size distribution measurements. Physical particle characteristics such as grain size distribution and sphericity are strongly linked to soil compaction, cohesion and shear strength, which makes it hard to evaluate these properties as separate values^19,22,39^. It is however an important consideration for lunar exploration. At shallow depths of regolith (<25–30 cm) excavation is not considered to be problematic for tools, however at deeper levels the bulk density increases owing to the interlocking of the regolith particles. Thus excavation will become more difficult – a case to highlight this was observed during Apollo 15 traverses where astronauts encountered a dense layer at 30–35 cm depth that could not be readily penetrated by their toolset and an improvised procedure had to be utilised.

For soils in the lunar particle size range, density and particle size and shape distribution exert a larger influence on mechanical properties than composition^20^. However, ISRU methods necessary for future human lunar habitation are receiving increasing attention. In order to correctly interpret the results gained from experiments with lunar regolith simulants, it is crucial to know the detailed characteristics of the material. Chemical and mineralogical variations within a simulant may give unexpected results during processes such as H_2_ reduction, oxide cracking and oxygen extraction, microwave sintering or metal extraction^34,40,41^.

EAC-1 shares major mineralogical components with lunar regolith samples from the Apollo missions, with the exception of the higher K_2_O and NaO wt% in EAC-1 alkali minerals (Fig. 4). Additionally, EAC-1 contains chlorite, quartz and feldspathoids, which are rare or completely absent in the lunar regolith^42^. Chlorites and feldspathoids contains crystalline water (HO^−^) that could affect processes such as regolith sintering or oxygen extraction. Variation in iron oxidation states could affect the magnetic properties of the regolith. EAC-1 contains ilmenite in the oxidized state as compared to the reduced version found in the lunar regolith, which formed in the absence of water and oxygen. The presence of nanophase iron in the lunar regolith could potentially be of importance, although this is less understood^7^, and only a few studies have tried to replicate this property^43^.

Carter *et al*.^44^ argued that the community should join forces to create a homogenous >100 tonnes host (“root”) simulant (both of mare and highland composition), which could consequently be developed into specialised simulants. A standardization (benchmarking) of lunar regolith simulants is important for reliable preparatory usefulness research based on physical, chemical and mineralogical properties^34,45^. Lunar regolith simulants should therefore include detailed classification of chemical and mineralogical components. For soils in the lunar particle size range, the density and the particle size and shape distribution exert a larger influence on mechanical properties than does composition^20^, so having a common set of grain size distribution mixes could also be beneficial to the lunar science community.

## Conclusion

The large-volume lunar regolith simulant EAC-1A shares similar physical characteristics to the lunar regolith, albeit with some notable deficiencies and variances. The physical properties of EAC-1A fall within the wide range of the lunar regolith with regards to cohesion, sphericity and grain size distribution. The major element composition of EAC-1 is comparable to the Apollo 17 samples with the main exception of the alkali components, feldspathoids and the hydrated amphibole and chlorite groups.These are common for terrestrial materials, but differ from lunar rock compositions. This has to be taken into consideration if EAC-1A is to be used for higher-fidelity ISRU purposes. The small grain size, the cohesive properties and the fact that it can be obtained at a low cost and in high quantities, means that EAC-1A fulfils many simulant requirements, in particular meeting the needs for the planned LUNA facility at EAC, Cologne.

## Supplementary information

Supplemental Information.
