# Supplementary material for: EAC-1A: A novel large-volume lunar regolith simulant
Source: Sci Rep. 2020 Mar 25;10:5473. doi: 10.1038/s41598-020-62312-4 (PMC7096503; doi:10.1038/s41598-020-62312-4)
Supplement: Supplementary file 1 — Supplemental Information. [file 41598_2020_62312_MOESM1_ESM.pdf]

EAC-1A: A novel large-volume lunar regolith simulant

V. S. Engelschiøn[1] , S. R, Eriksson[2], A. Cowley[2], M. Fateri[3], A. Meurisse[3], Ü. Kueppers[4] and M. Sperl[3], [1]The Natural History Museum, University of Oslo, Oslo, Norway, [2]European Astronaut Centre EAC, European Space Agency, 51170 Köln, Germany, [2] Institute for Materials Physics in Space, German Aerospace Center (DLR), 51147 Köln, Germany, [3]Department of Earth and Environmental Sciences, Ludwig-Maximilians-Universität (LMU) Munich, 80333 München, Germany.

Supplemental Information – Mineralogy of EAC-1

Normative mineralogy was calculated from the rock bulk chemistry for selected lunar regolith simulants and compared to the Königswinter basanite (see Table 1 – supplemental information).

Minerals	EAC-1	JSC-1A	CAS-1	FJS-1	Apollo 16
Quartz	-	-	-	4.27	0
Plagioclase	29.71	54.55	52.31	52.23	75.78
Orthoclase	6.15	4.73	6.09	5.97	0.95
Nepheline	5.08	-	-	-	0
Diposide	23.73	9.13	6.39	11.09	3.12
Hypersthene	-	2.05	10.89	12.59	6.77
Olivine	13.88	10.78	18.98	-	10.77
Ilmenite	0.45	0.43	3.36	3.63	0.97
Hematite	13.52	12.40	-	-	0
Apatite	1.51	1.62	0.70	1.02	0.25
Perovskite	3.48	-	-	-	0
Sphene	-	4.11	-	-	0
Magnetite	-	-	-	6.92	0
Total (wt%)	97.51	99.80	98.72	97.72	98.6

Supplemental Table 1. A computational estimate for mineralogy based on the bulk chemistry. EAC-1 is compared to available data from other simulants and an average of Apollo 16 samples (Kanamori et al., 1998; Zheng et al., 2009; Ray et al., 2010).

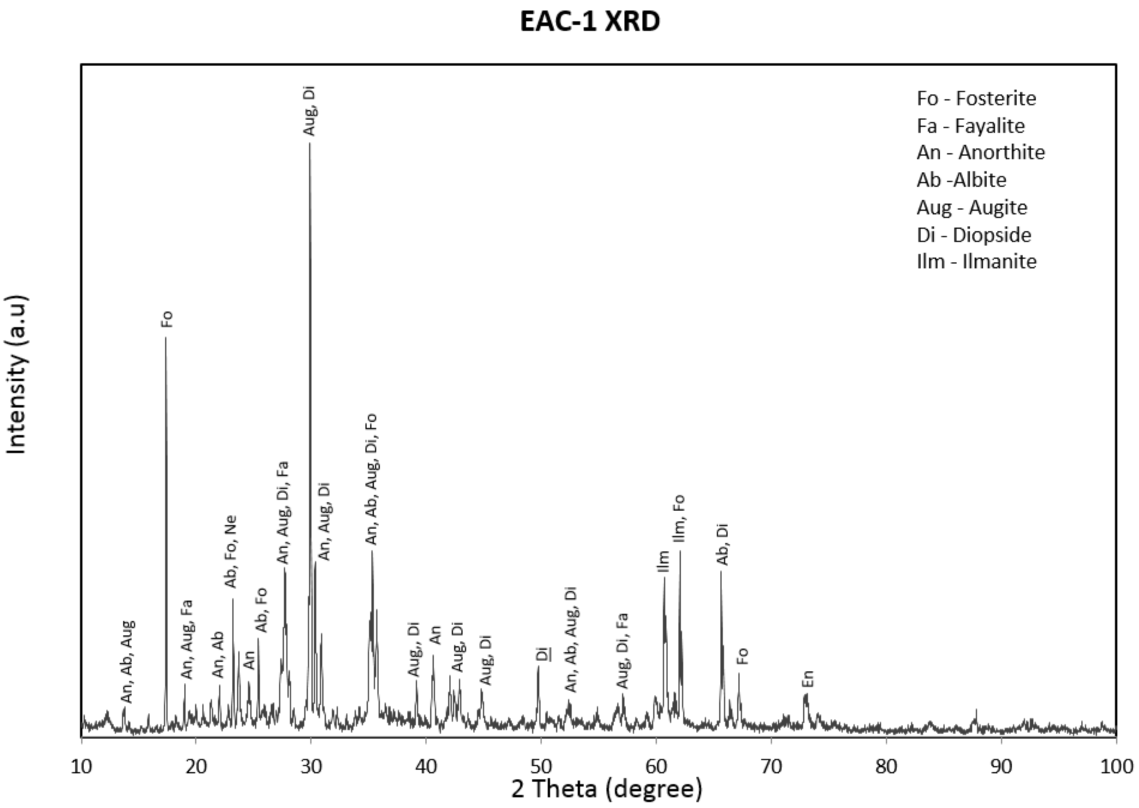

Supplemental Figure 1. XRD data obtained from EAC-1. Crystallographic mineral signatures were determined using the RUFF mineral reference database.

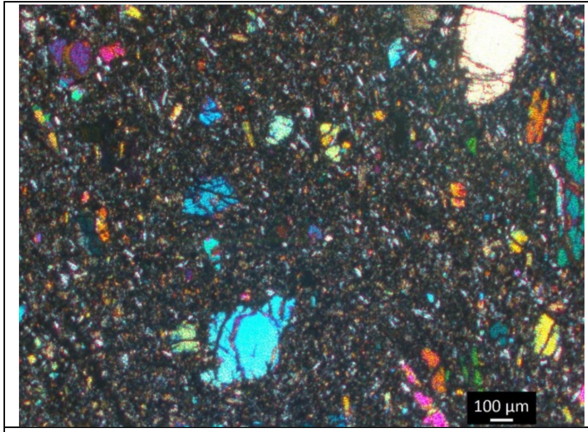

General picture of OP-1 including major minerals include olivine, pyroxene and plagioclase.

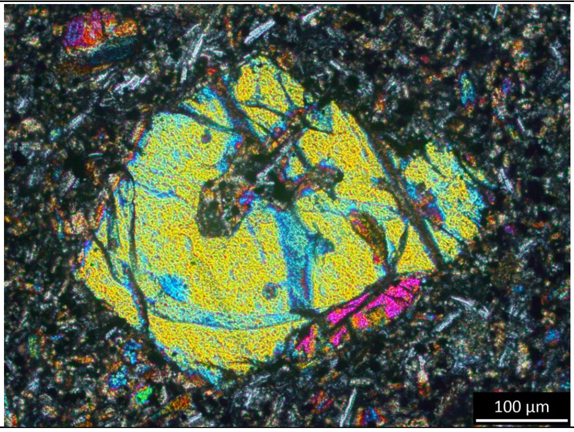

Typical olivine found in OP-1. The olivine cracked with serpentization filling the cracks.

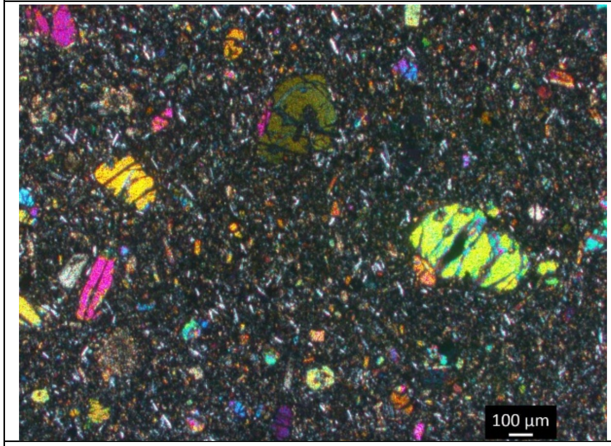

General picture representative of OP-1. Olivine can be seen clearly in the thin section including a characteristic matrix dominated by plagioclase.

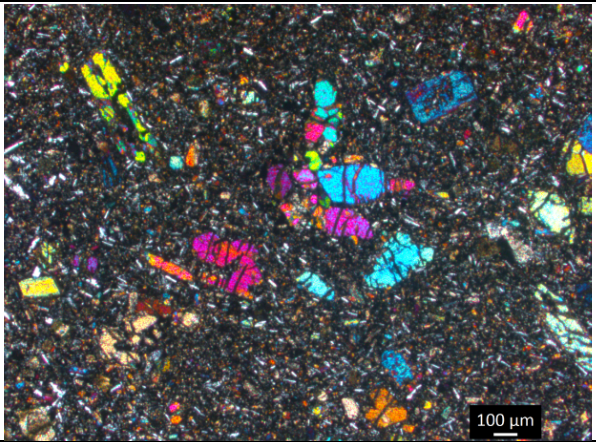

Overview picture of OP-1 including a larger olivine glomerocrysts.

Supplemental Figure 2. Additional microscopy presentation of the thin section OP-1.

	Mineral Content	Crystal Habit	Grain Size
Plagioclase	64 Volume %	Euhedral to suphedral.	>100 µm, one exception (130 µm).
Olivine	25 Volume %	Anhedral to suphedral, largely fragmented.	Found in both larger a smaller grain size fractions. Dominant mineral above 100 µm.
Pyroxene	5 Volume %	Anhedral to suphedral.	Mainly <100 µm, though some smaller crystals are present.
Amphibole	0.5 Volume %	Euhedral.	400 µm.
Quartz	0.5 Volume %	Anhedral.	130 µm.
Opaque	5 Volume %	Euhedral to suphedral.	>100 µm.

Supplemental Table 2. General petrographic overview of OP-1 based on thin section analysis.

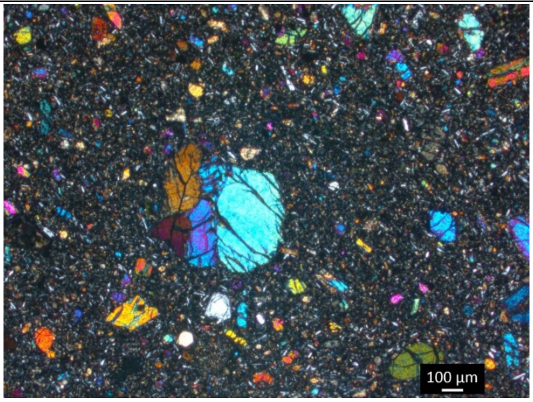	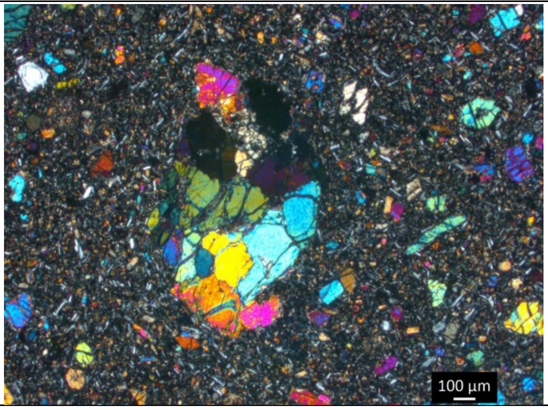
General picture of sample OR-1. The section clearly shows an olivine glomerocrysts and singular olivines. Plagioclase and pyroxene can also be seen.	Olivine glomerocrysts in OR-1.
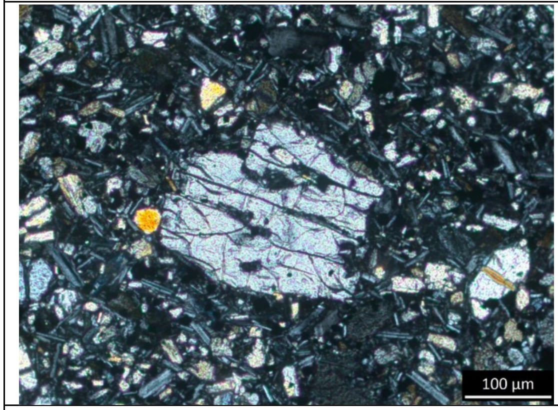	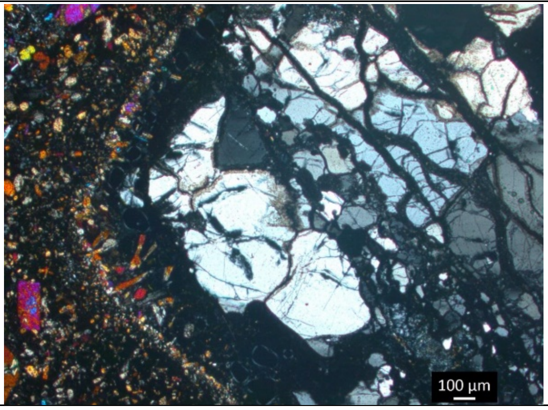
Plagioclase rich unit found in OR-1. Larger quartz crystal centered in the sample.	Quartz fragment in OR-1 thin section.

Supplemental Figure 3. Additional microscopy presentation of the thin section OR-1.

	Mineral Content	Crystal Habit	Grain Size
Plagioclase	54 Volume %	Euhedral to suphedral.	Mainly found in grain sizes below 100 µm. However one plagioclase is found to be 150 µm.
Olivine	29 Volume %	Anhedral to suphedral, largely fragmented. Glomerocrysts are common.	Both found in smaller and larger grain size fractions. Both bellow and above 100 µm.
Pyroxene	10 Volume %	Anhedral to suphedral.	Both bellow and above 100 µm.
Quartz	2 Volume %	Anhedral, though angular fragments exist.	Large fragment reaching 1 cm. However the most dominant grain size is <100 µm.
Amphibole	2 Volume %	Suphedral to anhedral.	> 100 µm.
Opaque	5 Volume %	Euhedral to suphedral.	<100 µm.

Supplemental Table 3. General petrographic overview of OR-1 based on thin section analysis.

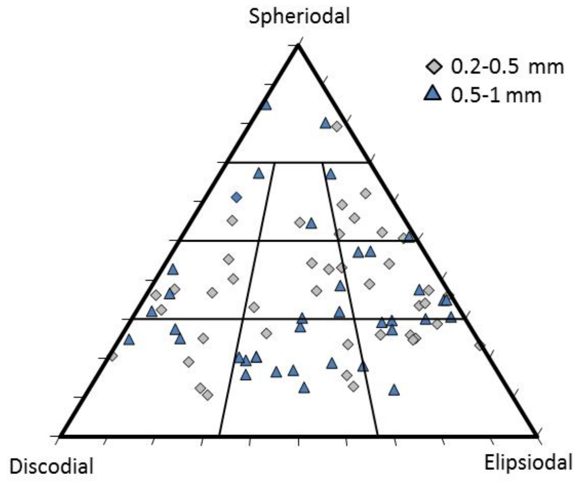

Supplemental Figure 4. Sphericity distribution of EAC-1 based on the mineral fractions 0.2 – 0.5 mm and 0.5 -1.0 mm. Both grain size fractions display a large variation in geometric character.

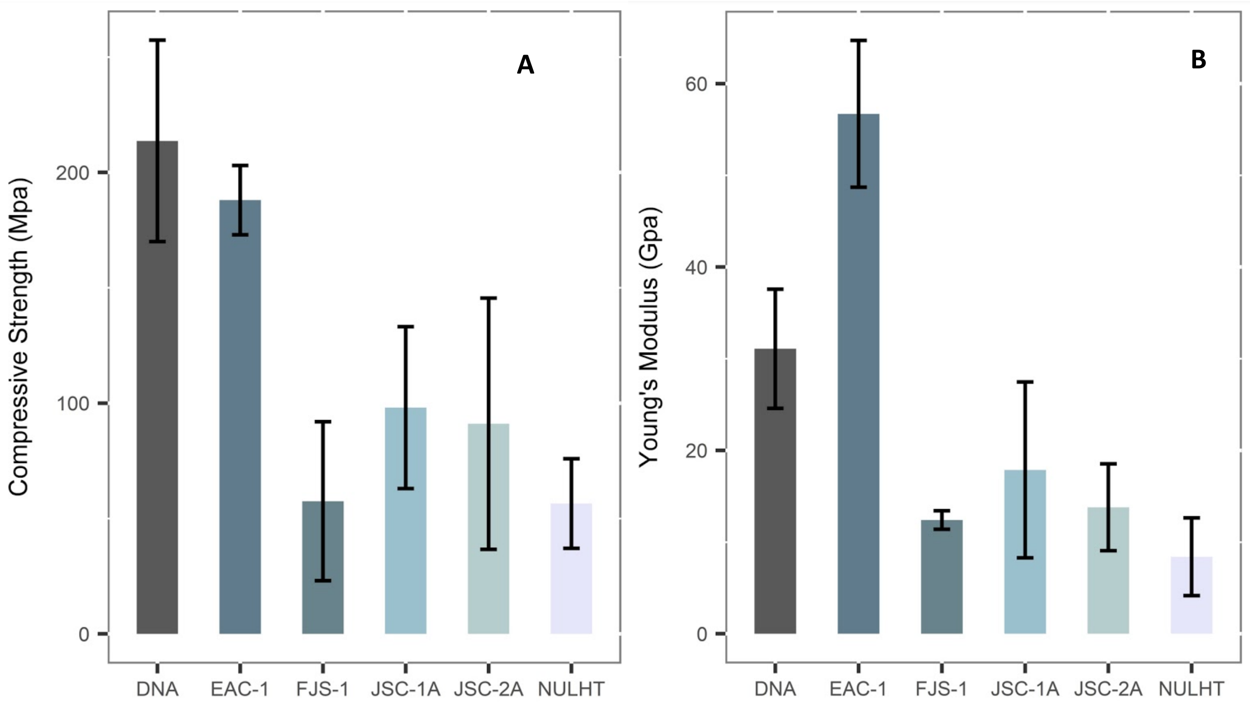

Supplemental Figure 5. Compressive strength of EAC-1 and other lunar regolith simulants are displayed in A. The Young's Modulus of EAC-1 and other lunar regolith simulants are displayed in B. Error bars are standard deviation across 3 samples

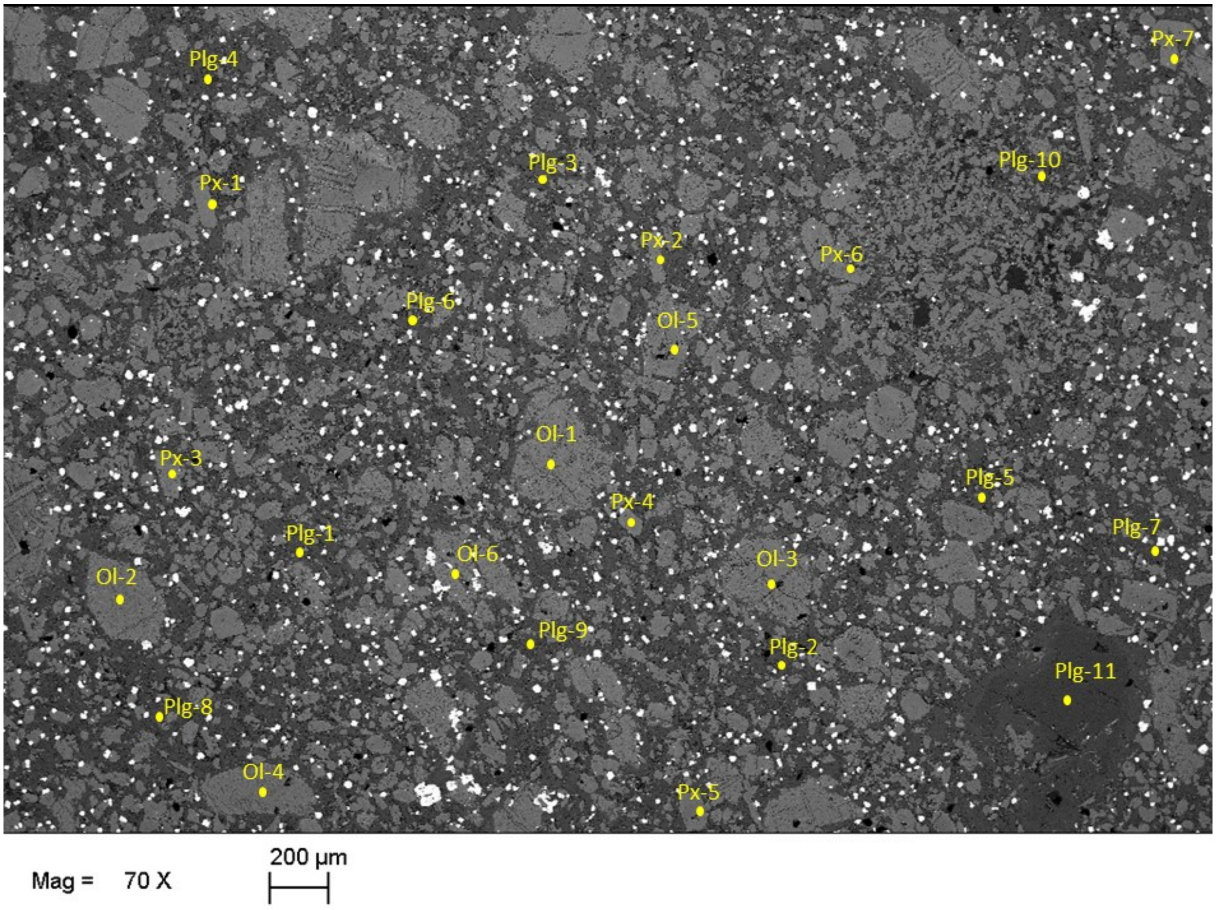

Supplemental Figure 6. SEM image of OR-1 sample, with marked EDX sample points and abbreviations corresponding to mineral type.

Sample Location	O	Na	Mg	Al	Si	P	S	K	Ca	Ti	Fe
Ol-1	42.21	0	20.67	0.82	18.25	0	0	0	0.30	0	17.38
Px-1	44.03	0.38	7.52	2.68	22.70	0	0	0	16.20	1.12	5.39
Plg-1	48.41	2.86	0	14.76	24.33	0	0	0.46	8.48	0.19	0.52

Supplemental Table 4. Whole surface and EDX spot measurement values from Sup. Fig. 6 representative of olivine, pyroxene and plagioclase composition in sample OR-1.

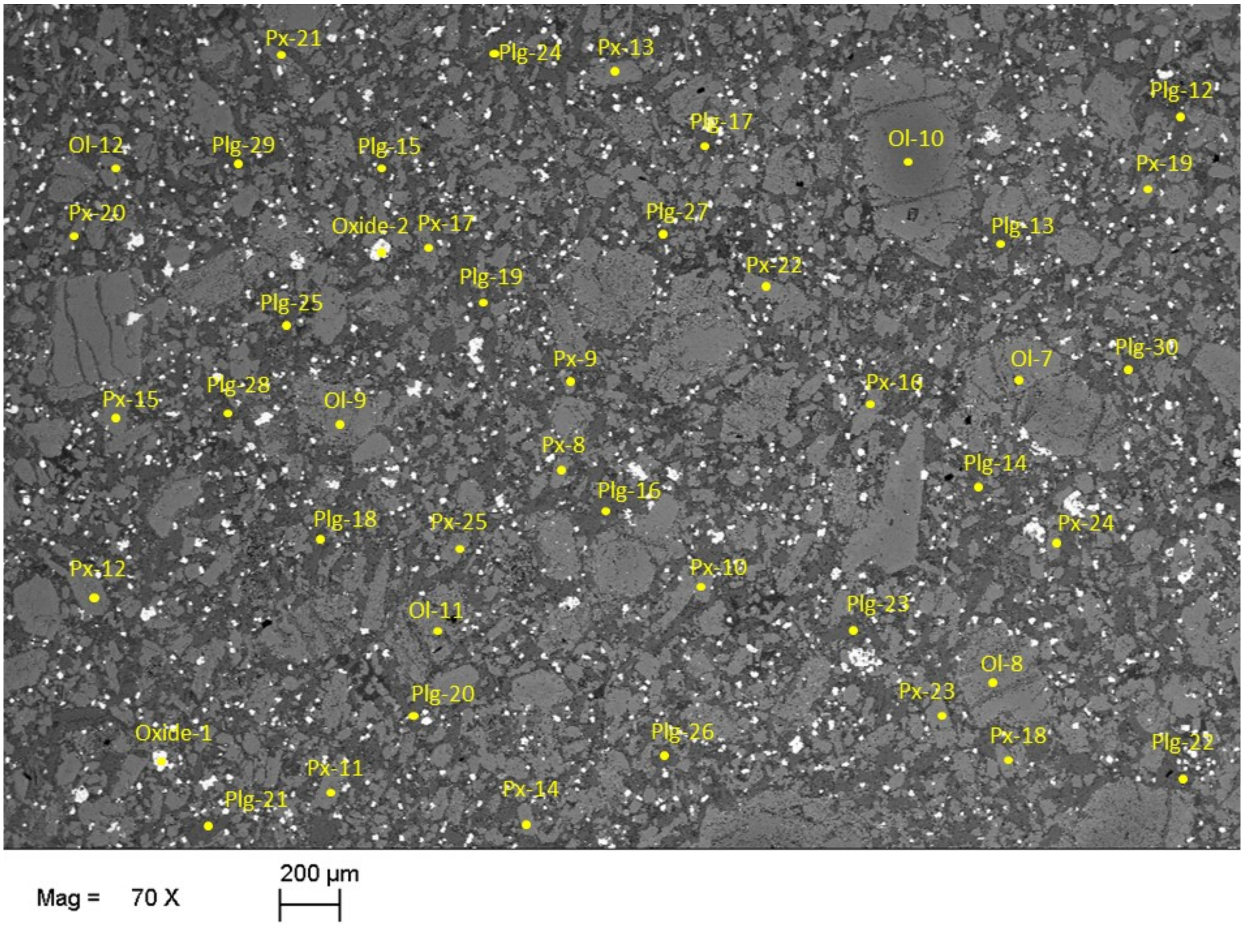

Supplemental Figure 7. SEM image of OP-1 sample, with marked EDX sample points and abbreviations corresponding to mineral type.

Sample Location	O	Na	Mg	Al	Si	P	S	K	Ca	Ti	Fe
Ol-11	44.19	0	23.68	0	18.1	0	0	0	0.19	0	14.31
Px-11	46.30	0.39	6.81	4.22	20.49	0	0	0	14.57	1.81	5.40
Plg-23	47.80	2.47	0	15.36	24.32	0	0	0.30	9.24	0	0.51

Supplemental Table 5. Whole surface and EDX spot measurement values from Sup. Fig. 6 representative of olivine, pyroxene and plagioclase composition in sample OP-1.
